# Robotic colorectal surgery in Latin America: a systematic review on surgical outcomes

**DOI:** 10.3389/fsurg.2024.1480444

**Published:** 2024-10-23

**Authors:** Bruno Augusto Alves Martins, Nicolas Avellaneda, Guglielmo Niccolò Piozzi

**Affiliations:** ^1^ Department of Colorectal Surgery, Hospital Universitário de Brasília, Brasilia, Brazil; ^2^ Department of General Surgery and Academic Investigations Unit, CEMIC University Hospital, Buenos Aires, Argentina; ^3^ Department of Colorectal Surgery, Portsmouth Hospitals University NHS Trust, Portsmouth, United Kingdom

**Keywords:** colorectal surgery, robotic surgical procedures, robotic training, minimally invasive surgical procedures, Latin America

## Abstract

**Background and objectives:**

Robotic approach in colorectal surgery is rapidly gaining interest, particularly in the context of rectal cancer resection. Despite economic barriers, substantial proliferation of robotic colorectal procedures has been observed throughout Latin America. However, there is a lack of data regarding intraoperative and early postoperative outcomes, as well as oncological and long-term results. This systematic review aims to provide an overview of the surgical outcomes of robotic-assisted colorectal approaches across Latin America.

**Material and methods:**

A systematic literature search of electronic databases, including PubMed, LILACS, Scopus, Cochrane Library and Scielo, was performed and reported in line with Preferred Reporting Items for Systematic Reviews and Meta-Analyses guidelines. The main target of the literature search was studies that reported outcomes of colorectal robotic surgery in Latin America.

**Results:**

A total of 9,694 published articles were identified from the initial search. Nine thousand six hundred thirty-six publications were excluded after title and abstract review and removal of duplicates. Fifty-eight articles were thoroughly reviewed, and 11 studies met the inclusion criteria. The critical appraisal of study quality (biases risk assessment) was performed according to the Joanna Briggs Institute (JBI) Manual for Evidence Synthesis. In general, the overall study quality was poor. Of the 11 studies included in the analysis, ten addressed intraoperative and early postoperative outcomes, seven addressed oncological/pathological outcomes, and just one addressed long-term outcomes. Ten studies evaluated intraoperative and early postoperative outcomes, encompassing a total of 425 patients, the majority of whom were diagnosed with colorectal cancer. Morbidity rates exhibited a range between 0% and 45.9%, while mortality ranged from 0% to 2.5%.

**Conclusion:**

Few studies have been published addressing intraoperative, postoperative, pathological, and oncological outcomes of robotic colorectal surgery in this region. Undoubtedly, there are unique challenges not encountered by developed countries, including economic obstacles in establishing structured training programmes and high-quality centres for the development of robotic surgery. Further studies are needed to assess the real extent of robotic surgery in the region and its results.

**Systematic Review Registration:**

https://www.crd.york.ac.uk/, PROSPERO (CRD42023494112).

## 1 Introduction

Minimally invasive surgery has improved outcomes following colorectal resections. Compared to the open approach, laparoscopy has demonstrated numerous advantages concerning short-term results, such as reduced postoperative pain, faster bowel and physical function recovery, shorter length of stay, and enhanced cosmesis (1). When considering long-term outcomes, laparoscopy is also associated with reduced incidence of small bowel obstruction, adhesion formation, and incisional hernias. Additionally, compared to open surgery, laparoscopic resection has no detrimental impact on oncological outcomes. These attributes have firmly established laparoscopy as the gold standard for colorectal resections (1).

In recent years, robotic-assisted colorectal procedures have gained interest, particularly in the context of rectal cancer resection. The recent REAL trial (robotic vs. laparoscopic surgery for middle and low rectal cancer: short-term outcomes of a multicentre randomised controlled trial) suggested that robotic surgery yields better short-term outcomes for middle and low rectal cancer than conventional laparoscopic surgery, including lower rates of positive circumferential margins, fewer conversions to open surgery, and a faster postoperative recovery (2). Conversely, the ROLARR and COLRAR trials failed to demonstrate the superiority of robotics over laparoscopy (3, 4). However, the ROLARR trial had sole significative bias due to comparing outcomes from expert laparoscopists vs. early robotic adopters with different stages in their learning curve, whilst the COLRAR was terminated prematurely because of poor accrual of data. Long-term results of the REAL trial and other multicentric RCTs are needed to confirm these results.

Robotic surgical platforms are designed to overcome the limitations inherent to laparoscopic surgery with straight instruments. Some technical advantages of robotic surgery include a stable and highly magnified three-dimensional visualisation, a surgeon-controlled environment, optimised ergonomic design, EndoWrist instruments allowing for seven degrees of freedom, motion scaling, and tremor filtering (5, 6).

Notwithstanding these potential advantages, the considerable cost remains a principal drawback of robotic surgery compared to conventional laparoscopic surgery (7). As is well-established with other technological advancements, this barrier frequently carries along disparities in healthcare access due to economic circumstances, particularly in lower and middle-income countries (LMICs) (8).

Latin America (LATAM) is a prominent example of an extremely wide geographical region wherein the paucity of financial resources carries along restricted access to cutting-edge technologies. To the best of our knowledge, the first robotic-assisted colorectal procedure in Latin America was conducted in Brazil in 2008 by Averbach and colleagues to treat deep infiltrating endometriosis with rectal involvement (9). Since then, a substantial proliferation of robotic colorectal procedures has been observed throughout LATAM. However, data regarding intraoperative and early postoperative outcomes and oncological and long-term results is scarce. On the other hand, the current development of new and more sustainable robotic platforms is increasing the interest for LMICs in adopting this novel approach.

Based on the identified research gap, this systematic review seeks to examine, assess, and summarize the current surgical results of robotic-assisted colorectal surgery in LATAM. The goal is to establish a foundation for effective policy enhancements in the field of colorectal robotic surgery. Specifically, the primary objective is to review research on the intraoperative and short-term outcomes of colorectal robotic surgeries conducted in LATAM. The secondary objective is to evaluate pathological and long-term outcomes for the subset of oncological procedures.

## 2 Materials and methods

### 2.1 Search strategy

The Preferred Reporting Items for Systematic Reviews and Meta-Analyses (PRISMA) guidelines have been followed when conducting and reporting the systematic review. The protocol was registered in the International Prospective Register of Systematic Reviews, PROSPERO, under registration number CRD42023494112. No ethical approval was obtained for this systematic review since the included data was retained from published reports.

Two authors (BAAM and NA) independently performed a systematic review of the English, Spanish, and Portuguese literature.

The following Medical Subject Heading [MeSH] terms were used either independently or matched with the Boolean operators “AND” or “OR”: “robotic surgical procedures”, “robotic-assisted surgery”, “colorectal surgery”, and “Latin America”.

The literature search included the following electronic databases: PubMed, Scopus, Cochrane Library, LILACS (Latin American and Caribbean Health Sciences Literature), and Scielo (Scientific Electronic Library Online). Published and ahead-of-publication studies dating from the inception of each database through July 31st, 2024, were screened. No specific language restrictions were applied. The reference lists from the selected studies were reviewed to identify any additional relevant studies.

### 2.2 Study selection, study outcomes, and data extraction

The study aimed to report studies evaluating perioperative and oncological outcomes of colorectal robotic surgeries performed in Latin America. Studies reporting colorectal robotic procedures in adults (age >18 years), regardless of the pathological condition or surgery indication (i.e., colon cancer, rectal cancer, diverticular disease, IBD, or endometriosis), were included in the analysis. There were no restrictions on the robotic surgical platform used in the procedures or the healthcare facility performing the procedure.

The primary outcome measures included operative time, conversion rate to laparotomy, length of hospital stay, postoperative morbidity, anastomotic leakage rate, and mortality. Secondary outcome measures included the number of harvested lymph nodes, positive surgical margins, quality of mesorectum excision, overall survival rate, and disease-free survival rate.

Study designs included randomized controlled trials and prospective and retrospective studies. The exclusion criteria were the following: (1) letters to the editor, (2) case reports, (3) video vignettes, (4) animal studies, and (5) non-available full-text articles. Studies without any defined clinical outcomes were also excluded.

Upon screening the preliminary records, we excluded duplicate reports and conference abstracts without a full text, after which the remaining articles were screened. An initial screening by title and abstract was performed, followed by a full-text screening of the selected articles to check for eligibility. Any disagreement was resolved by a third independent reviewer (GNP). Each included manuscript was read to determine ultimate inclusion in the final analysis. In the case of more than one study published by the same authors with overlapping data or periods, the study with a more adequate design was considered for the review.

From each manuscript, the following information was extracted: first author, year of publication, country, study design, number of patients included, operative time, conversion rate, length of stay, postoperative morbidity, anastomotic leakage rate, and mortality. Studies were also screened for information regarding long-term complications and oncological/pathological outcomes.

Missing data was reported as “NR” (not reported). An Excel database (version 15.21.1, Microsoft, Redmond, WA, USA) was used for data recording.

### 2.3 Methodological quality appraisal

The critical appraisal of study quality (biases risk assessment) was performed by the first author (BAAM) and revised by all the coauthors (NA and GNP) according to the Joanna Briggs Institute (JBI) Manual for Evidence Synthesis (10). No predetermined criteria for exclusion were defined. Any disagreement was resolved by a third independent reviewer (GNP).

The risk of bias was ranked as high when the study reached up to 49% of the “yes” score, moderate when the study reached 50% to 69% of the “yes” score, and low when the study reached over 70% of the “yes” score.

### 2.4 Data analysis and statistics

Given that the majority of the studies lacked a control group, a meta-analysis of the data was precluded. As such, the results from each study were presented in a summarised and aggregated form. Categorical data were expressed as absolute values and/or pooled percentages. Continuous data were expressed as absolute mean/median values with ranges.

## 3 Results

**Figure 1:**
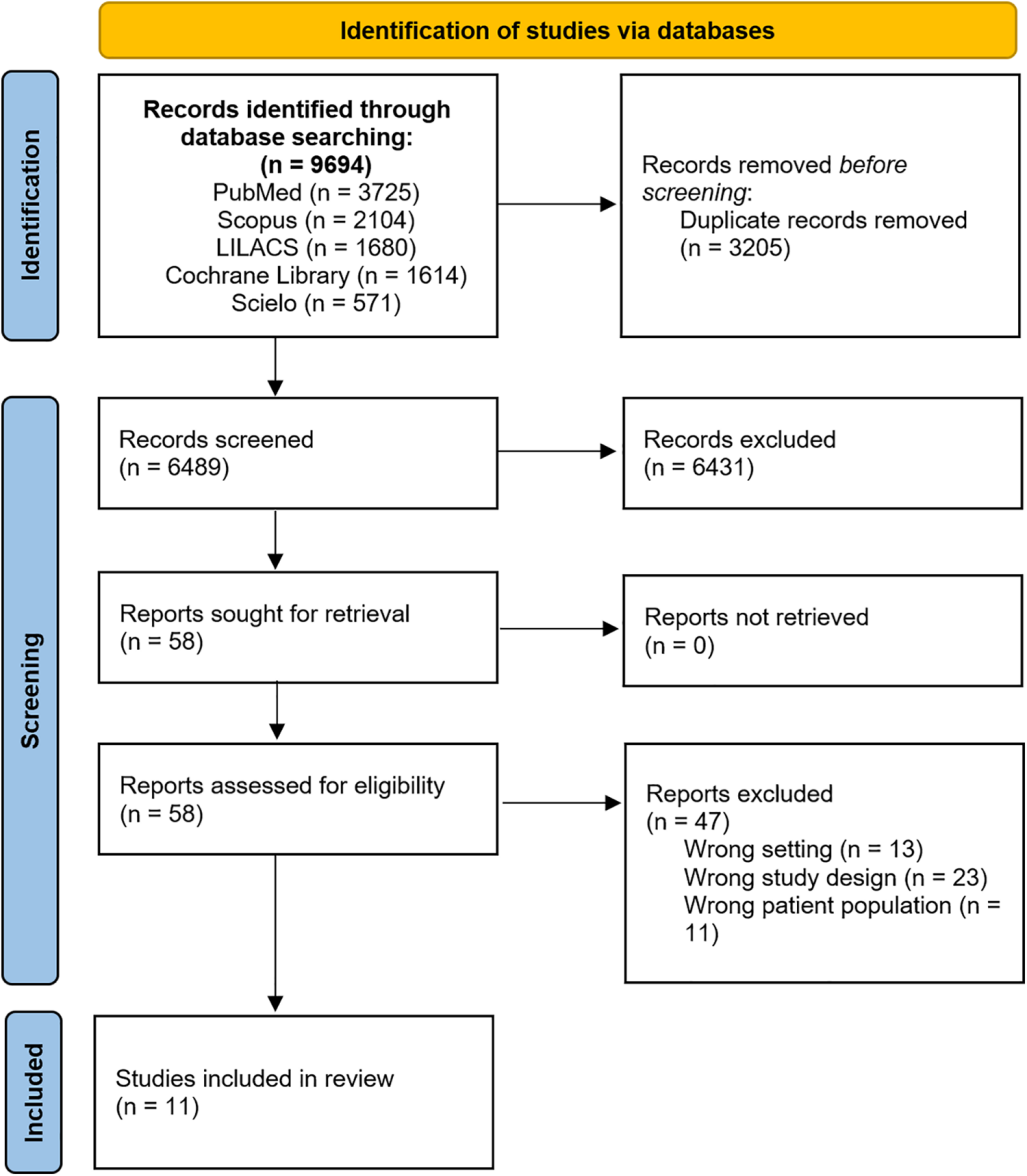
PRISMA flow chart illustrating the process of literature search and study selection. PRISMA, preferred reporting items for systematic reviews and meta-analyses.

A total of 9,694 published articles were identified from the initial search. Nine thousand six hundred thirty-six publications were excluded after title and abstract review and removal of duplicates. Fifty-eight articles were thoroughly reviewed, and 11 studies (11–21) met the inclusion criteria. Figure 1 demonstrates the PRISMA flow diagram.

Seven articles were produced in Brazil, two in Mexico, one in Argentina and one in Chile. Most studies were retrospective series. No randomised controlled trial was identified. Just one study presented a comparative analysis between robotic, laparoscopic and open approaches. Additionally, studies had a small sample size. Just two studies presented a sample size greater than 100 patients.

Of the 11 studies included in the analysis, ten addressed intraoperative and early postoperative outcomes, seven addressed oncological/pathological outcomes, and just one addressed long-term outcomes.

The methodological quality appraisal of the 11 studies included is described in Table 1. Generally, the overall study quality was poor due to their retrospective, registry-based nature. The risk of bias should be considered high in 5 of the 11 studies. In most studies, it was unclear whether case series had consecutive and complete inclusion of participants.

**Table 1:** Critical appraisal checklist for observational studies (Joanna Briggs institute) in the systematic review.

	Neme et al. (9)	Ramos et al. (10)	Villanueva-Sáenz et al. (11)	Madureira et al. (12)	Valadão et al. (13)	Lococo et al. (14)	Denadai et al. (15)	Morrell et al. (16)	López-Köstner et al. (17)	Guraieb-Trueba et al. (18)	De Jesus et al. (19)
1. Were there clear criteria for inclusion in the case series?	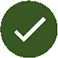	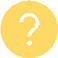	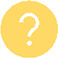	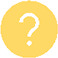	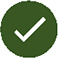	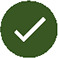	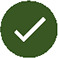	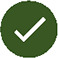	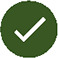	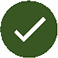	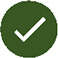
2. Was the condition measured in a standard, reliable way for all participants included in the case series?	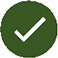	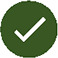	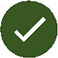	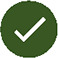	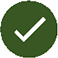	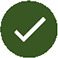	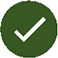	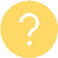	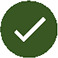	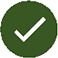	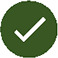
3. Were valid methods used for identification of the condition for all participants included in the case series?	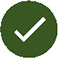	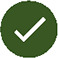	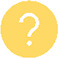	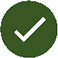	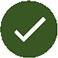	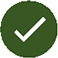	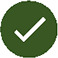	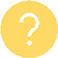	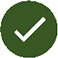	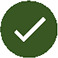	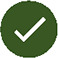
4. Did the case series have consecutive inclusion of participants?	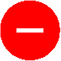	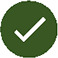	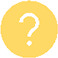	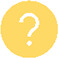	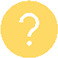	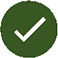	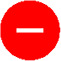	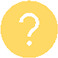	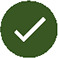	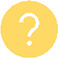	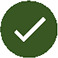
5. Did the case series have complete inclusion of participants?	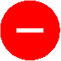	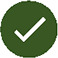	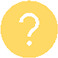	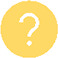	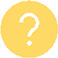	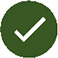	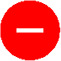	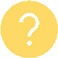	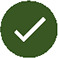	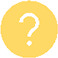	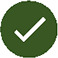
6. Was there clear reporting of the demographics of the participants in the study?	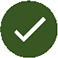	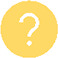	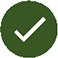	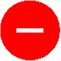	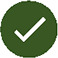	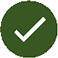	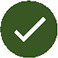	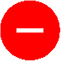	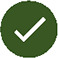	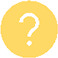	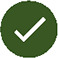
7. Was there clear reporting of clinical information of the participants?	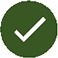	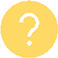	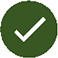	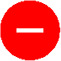	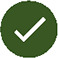	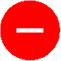	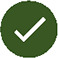	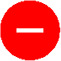	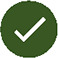	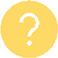	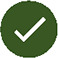
8. Were the outcomes or follow-up results clearly reported?	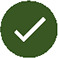	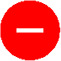	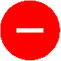	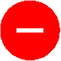	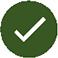	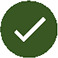	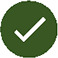	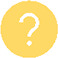	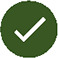	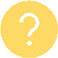	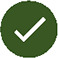
9. Was there clear reporting of the presenting site(s)/clinic(s) demographic information?	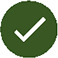	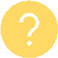	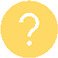	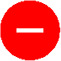	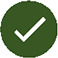	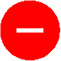	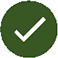	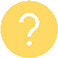	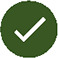	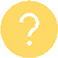	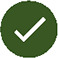
10. Was statistical analysis appropriate?	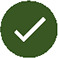	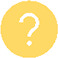	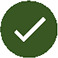	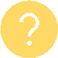	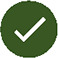	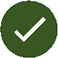	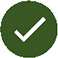	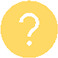	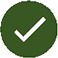	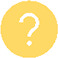	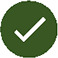
%Yes/risk of bias	80% (Low)	40% (High)	40% (High)	20% (High)	80% (Low)	80% (Low)	80% (Low)	10% (High)	100% (Low)	30% (High)	100% (Low)

Yes 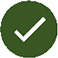; no: 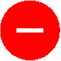; unclear: 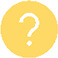. The risk of bias was ranked as high when the study reached up to 49% of the “yes” score, moderate when the study reached 50%–69% of the “yes” score, and low when the study reached over 70% of the “yes” score.

### 3.1 Intraoperative and early postoperative outcomes

Ten studies evaluated intraoperative and early postoperative outcomes. The baseline characteristics, such as first author, year of publication, country, study design, number of patients included, operative time, conversion rate, postoperative morbidity, and mortality, are summarised in Table 2. These studies encompass a total of 425 patients, the majority of whom were diagnosed with colorectal cancer. All surgeries were performed using the *da Vinci* robotic system (Intuitive Surgical Inc., Sunnyvale, CA, USA).

**Table 2:** Baseline characteristics from studies that evaluated early postoperative outcomes.

Author, year	Country	Study design	Patients (n)	Mean operative time (minutes)	Conversion rate	Morbidity	Anastomotic leakage rate	Length of stay (days)	Mortality
Neme et al. (11)	Brazil	Case series (retrospective)	10 patients with colorectal endometriosis	157	Nil	Nil	Nil	3 (mean)	Nil
Ramos et al. (12)	Brazil	Case series (prospective)	6 patients with mid/low rectal cancer	245	Nil	16%	Nil	6 (median)	Nil
Villanueva-Sáenz et al. (13)	Mexico	Retrospective	5 patients (3 with CRC, 1 with rectal prolapse, and 1 with diverticular disease	350	Nil	Nil	Nil	5 (mean)	Nil
Madureira et al. (14)	Brazil	Retrospective	59 (diagnosis not reported)	Not reported	Not reported	5%	1.7%	3.4 (mean)	Nil
Valadão et al. (15)	Brazil	Retrospective	117 patients with CRC	Not reported	1.7%	28%	11%	5 (median)	2.5%
Lococo et al. (16)	Argentina	Retrospective	41 (38 patients with neoplasia, 1 with rectal prolapse and 2 with diverticular disease)	170 (median)	7.3%	19.5%	9.75%	4 (median)	2.4%
Denadai et al. (17)	Brazil	Retrospective	102 patients with rectal cancer	273.9	1.93%	23.5%	3.9%	4.5 (mean)	1.9%
Morrell et al. (18)	Brazil	Retrospective	44 patients who underwent high or low anterior resections for benign and malignant diseases	Not reported	Nil	Nil	Nil	Not reported	Nil
López-Köstner et al. (19)	Chile	Prospective cohort	36 patients with rectal cancer	266	5.4%	45.9%	18.2%	9.6 (mean)	Nil
Guraieb-Trueba et al. (20)	Mexico	Case series (retrospective)	5 patients underwent R-TAMIS	85	Nil	Nil	Not applicable	1.6 (mean)	Nil

CRC, colorectal cancer.

Among the studies included, five reported no conversions to an open approach during the procedures performed, while one study did not provide this information. In the remaining studies, the conversion rate ranged from 1.7% to 7.3%. Regarding operative time, the measures of central tendency assessed in the studies indicated a range from 157 to 350 min. Concerning length of hospital stay, the measures of central tendency evaluated in the studies revealed a range from 1.6 to 9.6 days.

In relation to early postoperative outcomes, morbidity rates exhibited a range between 0% and 45.9%, while mortality ranged from 0% to 2.5%. Four studies reported no anastomotic leakage event. In one study, anastomotic leakage rate was not applicable because the subject addressed was robotic transanal excision. The anastomotic leakage rate ranged from 1.7% to 18.2% among the remaining five studies.

Among the ten studies examined regarding surgical outcomes, one presented findings from robotic transanal approaches. In this study, Guraieb-Trueba and colleagues (20) delineated the outcomes of five patients with rectal lesions who underwent robotic transanal minimally invasive surgery. There were no conversions, and the mean surgery time was 85 min.

### 3.2 Oncological and pathological outcomes

Seven studies analysed oncological and pathological outcomes. The baseline characteristics, such as first author, year of publication, country, study design, number of patients included, number of harvested lymph nodes, positive surgical margins, and quality of mesorectum excision, from studies that evaluated oncological and pathological outcomes, are summarised in Table 3.

**Table 3:** Baseline characteristics from studies that evaluated oncological and pathological outcomes.

Author, year	Country	Study design	Patients (*n*)	Number of harvested lymph nodes	Positive surgical margins	Quality of mesorectum excision
Ramos et al. (12)	Brazil	Case series (prospective)	6 patients with mid/low rectal cancer	22 (median)	Nil	Complete total mesorectal excision in 5 specimens and nearly complete in one
Valadão et al. (15)	Brazil	Retrospective	117 patients with CRC	Not evaluated	0.8%	Not evaluated
Lococo et al. (16)	Argentina	Retrospective	38 patients with CRC	14.65 (median)	2.63%	Not evaluated
Denadai et al. (17)	Brazil	Retrospective	102 patients with rectal cancer	15 (median)	4.9%	Not evaluated
López-Köstner et al. (19)	Chile	Prospective cohort	36 patients with rectal cancer	15 (mean)	Nil	18.4 mm (mean size of circumferential margin)
Guraieb-Trueba et al. (20)	Mexico	Case series (retrospective)	5 patients underwent R-TAMIS	Not applicable	Nil	Not applicable
De Jesus et al. (21)	Brazil	Prospective cohort	59 patients with distal rectal adenocarcinoma	10 (mean)	16.4%	6 mm (mean size of circumferential margin)

CRC, colorectal cancer; R-TAMIS, robotic transanal minimally invasive surgery.

Lymph node yield ranged from 10 to 22 according to the measures of central tendency evaluated in the studies. Three studies described no compromised surgical margins. The highest rate of positive surgical margins was described by De Jesus and collaborators (21), who evaluated outcomes from 59 patients with distal rectal adenocarcinoma who underwent robotic-assisted resection. They found a rate of 16.4% of involved circumferential resection margins.

Three studies evaluated the quality of mesorectum excision specimens. Ramos and collaborators (12) described that five out of six patients with distal rectal adenocarcinoma had a complete total mesorectal excision, and the last one had a nearly complete resection. The other two studies evaluated mesorectal excision quality by examining the circumferential margin's mean size. De Jesus and colleagues (21) found a mean size of 6 mm, while López-Köstner and colleagues (19) reported a mean size of 18.4 mm.

### 3.3 Long-term outcomes

López-Köstner and colleagues delineated long-term outcomes for 37 patients with rectal adenocarcinoma who underwent robotic-assisted surgery. The mean follow-up period extended to 21 months, revealing a 100% overall survival rate and disease-free survival rate. Nearly 88% of the patients underwent stoma closure during the follow-up period (19).

## 4 Discussion

Latin American countries are witnessing a notable implementation of robotic colorectal surgery despite socioeconomic constraints and the high cost associated with acquiring and maintaining robotic platforms. In Brazil, for instance, 106 da Vinci (Intuitive Surgical Inc., Sunnyvale, CA, USA) robotic systems had been installed by 2023. However, a noticeable discrepancy exists in the distribution of this technology, with most robotic systems only accessible to private institutions and larger economic centres (22). By 2017, only ten public hospitals across Latin America had acquired robotic systems, and half of the institutions had their programmes temporarily or definitively interrupted, mainly due to the high costs of disposable instruments (23). As a result, a large portion of the population reliant on the public healthcare system faces considerable delays in accessing advanced healthcare technologies like robotic surgery. Economic barriers also hinder the development of structured training programs, leading to a shortage of specialized surgeons, a lack of standardized surgical training, and network issues that contribute to disparities between different socioeconomic settings. To date, in Latin America, the training process has been largely driven by the industry and private institutions that have adopted robotic surgical platforms (22).

This systematic review has revealed that the currently available data on surgical outcomes are limited and of poor quality. No randomized controlled trials were identified in the literature search, and even the retrospective series had limitations, such as a reduced number of patients and the absence of multicentric studies. Additionally, just one study has addressed long-term oncological outcomes, limiting the ability to draw conclusions about the broader impact of robotic surgery on survival and recurrence.

Moreover, from the included studies, only one (21) has conducted a comparative analysis with laparoscopic and open approaches. When discussing robotic abdominal procedures, it's important to compare them with the conventional approaches. This is particularly crucial in low- and middle-income countries, where we need to consider the costs and benefits to assess whether the adoption of robotic platforms is cost-effective.

Despite the methodological limitations, the findings of the existing studies demonstrate that robotic colorectal surgery in Latin America seems to be safe and feasible when it comes to perioperative and oncological outcomes. Notably, low conversion rates to open surgery and reasonable operative times were identified. Morbidity and mortality rates were also comparable to the data reported in the literature (24). A relevant sign of surgical oncological quality is the harvested lymph node rate, which ranges from 10 to 22. Khajeh and colleagues recently conducted a meta-analysis on the outcomes of robotic rectal cancer surgery. The number of excised lymph nodes was reported in 15 studies comprising 3,084 patients (1,569 patients in the robotic and 1,515 patients in the laparoscopic group). The mean number of harvested lymph nodes ranged from 10.3 to 25.5 in the robotic group, and the pooled analysis showed no significant difference compared to the laparoscopic group (24).

Regarding surgical margins, one study from LATAM found a rate of 16.4% of involved circumferential resection margins among 59 patients with distal rectal adenocarcinoma who underwent robotic-assisted resection. In comparison with open (15.8%) and laparoscopic (15.5%) approaches, there was no statistically significant difference (21). These results should be interpreted with caution since the data came from a single institution, and the robotic group comprises unselected consecutive patients who were operated on by three surgeons in their initial experience with the robotic approach. The impact of the learning curve should be emphasized, highlighting the early experiences of the Latin-American institutions. Therefore, it is currently not appropriate to directly compare the outcomes with those from well-established techniques, such as laparoscopy, or from centres of excellence in East Asia or Europe, but it is relevant for investigating the current trend in LATAM.

Evidence coming from Europe, the USA, China, Australia and India describing the initial experience of robotic colorectal surgery has been showing reduced conversion rates to open surgery, comparable short-term outcomes to conventional laparoscopic surgery, and adequate pathological and oncological outcomes (25–29). This kind of data suggests that Latin America is charting a comparable course. Undoubtedly, there are unique challenges not encountered by developed countries, including economic obstacles in establishing structured training programmes and high-quality centres for the development of robotic surgery. Nonetheless, the emerging data from Latin American institutions indicates that robotic colorectal surgery appears to be both safe and feasible.

To the best of our knowledge, this study represents the very first analysis of outcomes associated with robotic colorectal surgery throughout LATAM. A comprehensive literature search was conducted, encompassing key Latin American databases. As mentioned earlier, the principal limitations of this study are associated with the generally low quality of the included articles. Included papers were, for the most part, either retrospective or involved retrospective analysis of a prospectively maintained database. Most of the case series also derived from single-institution experiences with small patient cohorts, considerably impairing the sample's representativeness. There is also a lack of critical discussion of confounding factors. Furthermore, the absence of identified randomised controlled trials is noteworthy. Additionally, there is significant heterogeneity among the included papers, blending analyses of both oncological and benign conditions. Therefore, it was not possible to synthesise the results or carry out a meta-analysis as the studies were clearly heterogeneous. Overall, the study provides valuable insight into robotic colorectal surgery in LATAM, but the quality and variability of the available evidence constrain the conclusions. Higher-quality studies, especially randomized controlled trials and multi-institutional analyses with larger cohorts, will be needed to provide more definitive conclusions.

## 5 Conclusion

Latin America has witnessed an increasing rate of robotic surgical platforms installed, mainly within private institutions and large socioeconomic centres. In the domain of colorectal surgery, few studies have been addressing the intraoperative, postoperative, pathological, and oncological outcomes of this approach in this region. More studies are needed to assess the real extent of robotic surgery in the region and its results. Future research should concentrate on augmenting high-quality data concerning both short-term and long-term outcomes of the implementation of this technology. Additionally, comparisons with established techniques, such as laparoscopic approaches, should be explored to further enhance the understanding of the technology's efficacy, applications, and limitations.

## 6 Future directions

Healthcare systems across Latin America continue to face substantial structural problems and economic hurdles. Despite these challenges, surgical leaders and health managers must spearhead efforts to establish structured programmes guiding the adoption and training of robotic colorectal surgery. Centres of high-quality healthcare should play a central role in disseminating knowledge and preparing surgeons and other healthcare professionals to propagate new technologies and techniques throughout the countries. Creating a regional database is also imperative for recording clinical and surgical outcomes. Moreover, international collaboration could facilitate the establishment of high-quality centres and support the development of randomized controlled trials and multi-institutional analyses. Through this initiative, LATAM can generate auditable data, fostering a cycle of improvement. Anticipated technological advancements and increased market competition are expected to lead to cost reductions, thereby facilitating a more widespread deployment of robotic platforms and the subsequent democratisation of access. The expanding use of 5G communication technology opens opportunities for telesurgery and telementoring, enabling experienced surgeons to supervise another surgeon or trainee in a remote location. This tool can contribute significantly to delivering high-quality healthcare to remote and isolated communities in a region with vast geographical dimensions like LATAM.

## Data Availability

The original contributions presented in the study are included in the article/Supplementary Material, further inquiries can be directed to the corresponding author.
